# 6-(4-Meth­oxy­phen­yl)naphtho[2,3-*b*][1]benzothio­phene

**DOI:** 10.1107/S1600536812047137

**Published:** 2012-11-24

**Authors:** V. Silambarasan, T. Srinivasan, R. Sivasakthikumaran, A. K. Mohanakrishnan, D. Velmurugan

**Affiliations:** a CAS in Crystallography and Biophysics, University of Madras, Guindy Campus, Chennai-25, India; b Department of Organic Chemistry, University of Madras, Guindy Campus, Chennai-25, India

## Abstract

The asymmetric unit of the title compound, C_23_H_16_OS, contains two independent mol­ecules with opposite orientations of the meth­oxy groups bonded to the benzene rings. The napthobenzothiophene group in the two molecules is separated by an average distance of 3.912 Å. In both mol­ecules, the napthobenzothio­phene unit is almost planar, with r.m.s deviations of 0.0522 and 0.0143 Å. The meth­oxy­phenyl ring makes dihedral angles of 67.0 (6)° and 70.4 (6)° with respect to the napthobenzothio­phene ring system in the two mol­ecules. The crystal packing features C—H⋯S, π–π [centroid–centroid distances = 3.666 (10) and 3.658 (10) Å] and C–H⋯π inter­actions, forming a sheet running along the *b*-axis direction.

## Related literature

For the biological activity of thio­phene derivatives, see: Bonini *et al.* (2005 ▶); Brault *et al.* (2005 ▶); Isloora *et al.* (2010 ▶); Xia *et al.* (2010 ▶). For a related structure, see: Gunasekaran *et al.* (2010 ▶).
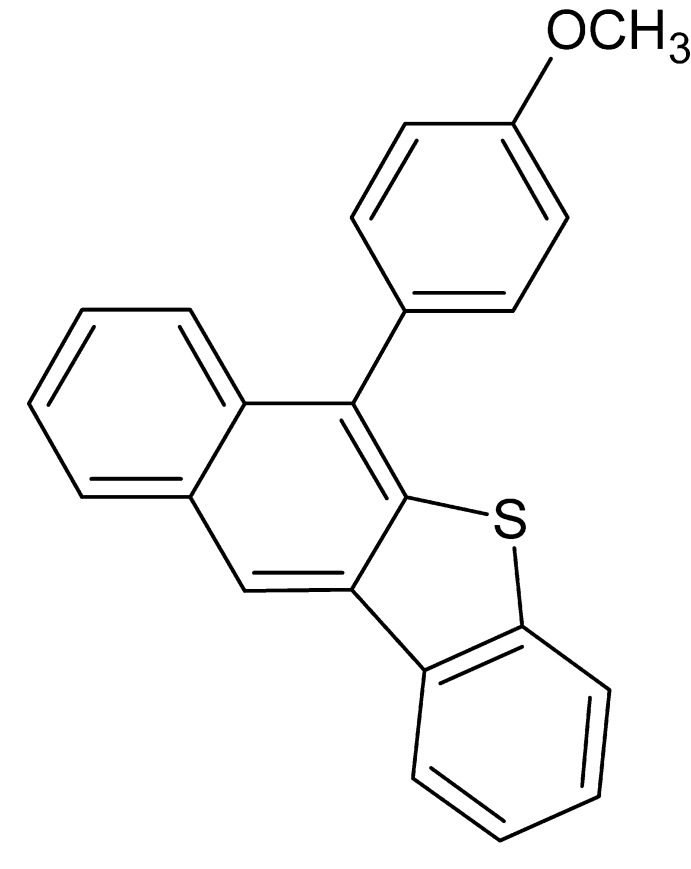


## Experimental

### 

#### Crystal data

C_23_H_16_OS
*M* *_r_* = 340.42
Triclinic, 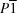

*a* = 6.2019 (3) Å
*b* = 11.2124 (6) Å
*c* = 24.4724 (13) Å
α = 95.759 (3)°
β = 91.762 (3)°
γ = 95.617 (3)°
*V* = 1683.63 (15) Å^3^
*Z* = 4
Mo *K*α radiation
μ = 0.20 mm^−1^
*T* = 293 K
0.20 × 0.20 × 0.20 mm

#### Data collection

Bruker SMART APEXII area-detector diffractometer
Absorption correction: multi-scan (*SADABS*; Bruker, 2008 ▶) *T*_min_ = 0.981, *T*_max_ = 0.985
30236 measured reflections
8440 independent reflections
6029 reflections with *I* > 2σ(*I*)
*R*_int_ = 0.041

#### Refinement

*R*[*F* ^2^ > 2σ(*F* ^2^)] = 0.046
*wR*(*F* ^2^) = 0.140
*S* = 1.03
8440 reflections
451 parameters
H-atom parameters constrained
Δρ_max_ = 0.37 e Å^−3^
Δρ_min_ = −0.28 e Å^−3^

### 

Data collection: *APEX2* (Bruker, 2008 ▶); cell refinement: *SAINT* (Bruker, 2008 ▶); data reduction: *SAINT*; program(s) used to solve structure: *SHELXS97* (Sheldrick, 2008 ▶); program(s) used to refine structure: *SHELXL97* (Sheldrick, 2008 ▶); molecular graphics: *ORTEP-3* (Farrugia, 2012 ▶); software used to prepare material for publication: *SHELXL97* and *PLATON* (Spek, 2009 ▶).

## Supplementary Material

Crystal structure: contains datablock(s) global, I. DOI: 10.1107/S1600536812047137/pv2598sup1.cif

Structure factors: contains datablock(s) I. DOI: 10.1107/S1600536812047137/pv2598Isup2.hkl

Additional supplementary materials:  crystallographic information; 3D view; checkCIF report

## Figures and Tables

**Table 1:** Hydrogen-bond geometry (Å, °) *Cg*2, *Cg*5 and *Cg*15 are the centroids of the C2–C7, C18–C23 and C9′–C14′ rings, respectively.

*D*—H⋯*A*	*D*—H	H⋯*A*	*D*⋯*A*	*D*—H⋯*A*
C6′—H6′⋯S1′^i^	0.93	2.87	3.7591 (17)	160
C3′—H3′⋯*Cg*15^ii^	0.93	2.93	3.704 (2)	141
C7—H7⋯*Cg*5^iii^	0.93	2.89	3.672 (2)	143
C11—H11⋯*Cg*2^iv^	0.93	2.91	3.789 (2)	159

Symmetry codes: (i) 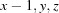; (ii) 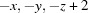; (iii) 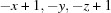; (iv) 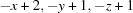.

**Fig. 1.**
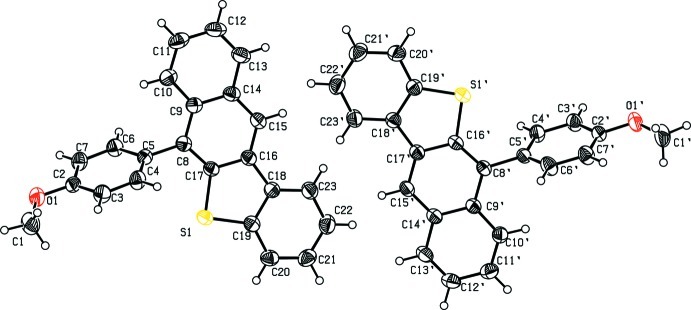
The molecular structure of the title compound with the atom numbering scheme. Displacement ellipsoids are drawn at the 30% probability level. H atoms are presented as small spheres of arbitrary radius.

**Fig. 2.**
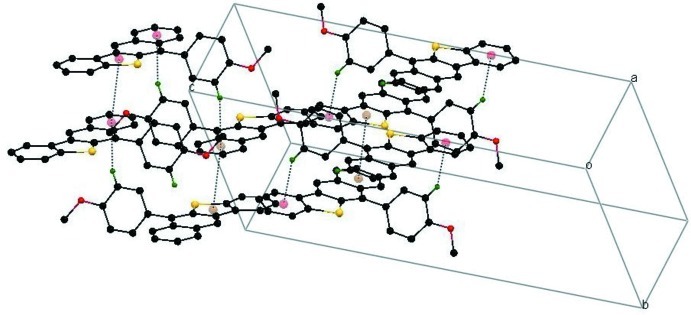
A view of the C—-H···π interactions (dotted lines) in the crystal structure of the title compound. H atoms non-participating in hydrogen-bonding were omitted for clarity.

## supplementary crystallographic information

### Comment

Thiophene derivatives exhibit anti-Human immunodeficiency virus protease (HIVPR) (Bonini *et al.*, 2005) and anti-breast cancer (Brault *et al.*, 2005) activities. In addition, some of the benzo[*b*]thiophene derivatives show significant antimicrobial and anti-inflammatory activities (Isloora *et al.*, 2010). The thiophene derivatives have been viewed as significant compounds for application in many fields (Xia *et al.*, 2010). In order to obtain detailed information on molecular conformations in the solid state, X-ray crystallographic study of the title compound was carried out.

An asymmetric unit of the title compound contains two crystallographically independent molecules with opposite orientations of the methoxy groups (Fig. 1). The bond lengths and angles agree with those observed in another benzothiophene derivative (Gunasekaran *et al.*, 2010). In both the molecules, napthobenzothiophene ring system is essentially planar, with maximum deviation of 0.103 (2) and 0.024 (1) Å for atoms C21 and S1', respectively. In both molecules, the napthobenzothiophene ring system (S1/C8—C23 and S1'/C8'-C23') makes dihedral angles of 67.0 (6)° and 70.4 (6)° with respect to benzene rings (C2–C7) and (C2'–C7'), respectively; showing that both the ring system are almost perpendicular to each other.

The π···π electron interactions are observed between the thiophene ring (S1/C16—C19 [at *x*, *y*, *z*] and the benzene ring (C9—C14) [at -1 + *x*, *y*, *z*] with centroid-centroid distance 3.666 (10) Å and between the thiophene ring (S1'/C16'–C19' [at *x*, *y*, *z*] and the benzene ring (C9'–C14') [at 1 + *x*, *y*, *z*] with the centroid-centroid distance 3.658 (10) Å. In addition the crystal packing is stabilized by C–H···S and C–H···π (Table. 1) types of interactions.

### Experimental

Benzo[b]thiophen-3-yl(2-((4-methoxyphenyl)pivaloyloxy)methyl)phenyl)methyl pivalate (0.73 g, 1.60 mmol) upon interaction with ZnBr_2_ (0.02 g, 0.13 mmol) followed by removal of solvent and column chromatographic purification (silica gel; hexane-ethyl acetate, 99:1) gave the compound as a colorless solid (0.50 g, 72%); the compound was recrystalized from chloroform.

### Refinement

All H atoms were fixed geometrically and allowed to ride on their parent C atoms, with C—H distances 0.93 and 0.96 Å for aryl and methyl H-atoms with *U*_iso_(H) = 1.5*U*_eq_(methyl-C) and 1.2*U*_eq_(aryl-C).

### Crystal data

**Table:**

C_23_H_16_OS	*Z* = 4
*M**_r_* = 340.42	*F*(000) = 712
Triclinic, *P*1	*D*_x_ = 1.343 Mg m^−^^3^
Hall symbol: -P 1	Mo *K*α radiation, λ = 0.71073 Å
*a* = 6.2019 (3) Å	Cell parameters from 8440 reflections
*b* = 11.2124 (6) Å	θ = 0.8–28.5°
*c* = 24.4724 (13) Å	µ = 0.20 mm^−^^1^
α = 95.759 (3)°	*T* = 293 K
β = 91.762 (3)°	Block, colourless
γ = 95.617 (3)°	0.20 × 0.20 × 0.20 mm
*V* = 1683.63 (15) Å^3^	

### Data collection

**Table:**

Bruker SMART APEXII area-detector diffractometer	8440 independent reflections
Radiation source: fine-focus sealed tube	6029 reflections with *I* > 2σ(*I*)
Graphite monochromator	*R*_int_ = 0.041
ω and φ scans	θ_max_ = 28.5°, θ_min_ = 0.8°
Absorption correction: multi-scan (*SADABS*; Bruker, 2008)	*h* = −8→8
*T*_min_ = 0.981, *T*_max_ = 0.985	*k* = −14→15
30236 measured reflections	*l* = −32→32

### Refinement

**Table:**

Refinement on *F*^2^	Primary atom site location: structure-invariant direct methods
Least-squares matrix: full	Secondary atom site location: difference Fourier map
*R*[*F*^2^ > 2σ(*F*^2^)] = 0.046	Hydrogen site location: inferred from neighbouring sites
*wR*(*F*^2^) = 0.140	H-atom parameters constrained
*S* = 1.03	*w* = 1/[σ^2^(*F*_o_^2^) + (0.0729*P*)^2^ + 0.216*P*] where *P* = (*F*_o_^2^ + 2*F*_c_^2^)/3
8440 reflections	(Δ/σ)_max_ = 0.001
451 parameters	Δρ_max_ = 0.37 e Å^−^^3^
0 restraints	Δρ_min_ = −0.28 e Å^−^^3^

### Special details

**Table:**

Geometry. All e.s.d.'s (except the e.s.d. in the dihedral angle between two l.s. planes) are estimated using the full covariance matrix. The cell e.s.d.'s are taken into account individually in the estimation of e.s.d.'s in distances, angles and torsion angles; correlations between e.s.d.'s in cell parameters are only used when they are defined by crystal symmetry. An approximate (isotropic) treatment of cell e.s.d.'s is used for estimating e.s.d.'s involving l.s. planes.
Refinement. Refinement of *F*^2^ against ALL reflections. The weighted *R*-factor *wR* and goodness of fit *S* are based on *F*^2^, conventional *R*-factors *R* are based on *F*, with *F* set to zero for negative *F*^2^. The threshold expression of *F*^2^ > σ(*F*^2^) is used only for calculating *R*-factors(gt) *etc*. and is not relevant to the choice of reflections for refinement. *R*-factors based on *F*^2^ are statistically about twice as large as those based on *F*, and *R*- factors based on ALL data will be even larger.

### Fractional atomic coordinates and isotropic or equivalent isotropic displacement parameters (Å^2^)

**Table:**

	*x*	*y*	*z*	*U*_iso_*/*U*_eq_	
S1	0.18748 (7)	0.11396 (4)	0.544498 (16)	0.04374 (13)	
S1'	0.32902 (7)	0.37462 (4)	0.961647 (16)	0.04379 (13)	
O1	0.4892 (2)	0.28907 (12)	0.32371 (5)	0.0549 (3)	
O1'	−0.0436 (2)	0.23774 (13)	1.18636 (5)	0.0575 (3)	
C17	0.4236 (2)	0.19074 (14)	0.57740 (6)	0.0373 (3)	
C8'	−0.0574 (2)	0.22460 (14)	0.95470 (6)	0.0366 (3)	
C18	0.2493 (3)	0.09979 (14)	0.65053 (6)	0.0400 (4)	
C16'	0.1203 (2)	0.27619 (14)	0.87037 (6)	0.0379 (3)	
C9	0.7591 (2)	0.31808 (14)	0.58557 (7)	0.0403 (4)	
C5'	−0.0552 (2)	0.23189 (14)	1.01597 (6)	0.0365 (3)	
C17'	0.1090 (2)	0.28311 (14)	0.92850 (6)	0.0363 (3)	
C8	0.5807 (2)	0.25893 (14)	0.55212 (6)	0.0385 (3)	
C18'	0.3128 (3)	0.34510 (14)	0.85358 (6)	0.0396 (4)	
C16	0.4363 (2)	0.17469 (14)	0.63429 (6)	0.0391 (3)	
C19	0.1000 (3)	0.06293 (14)	0.60615 (6)	0.0405 (4)	
C14'	−0.2168 (3)	0.14796 (14)	0.86256 (6)	0.0396 (4)	
C2	0.5044 (3)	0.28681 (15)	0.37968 (7)	0.0409 (4)	
C15'	−0.0416 (3)	0.20851 (15)	0.83843 (6)	0.0416 (4)	
H15'	−0.0352	0.2027	0.8003	0.050*	
C15	0.6110 (3)	0.22988 (15)	0.66590 (7)	0.0443 (4)	
H15	0.6216	0.2192	0.7031	0.053*	
C4'	0.0925 (3)	0.17389 (16)	1.04479 (6)	0.0449 (4)	
H4'	0.1928	0.1314	1.0256	0.054*	
C5	0.5587 (2)	0.27062 (14)	0.49206 (6)	0.0376 (3)	
C19'	0.4407 (3)	0.40253 (15)	0.89885 (7)	0.0417 (4)	
C4	0.3946 (3)	0.33000 (17)	0.47121 (7)	0.0495 (4)	
H4	0.3005	0.3652	0.4953	0.059*	
C9'	−0.2270 (2)	0.15599 (14)	0.92128 (6)	0.0377 (3)	
C7	0.6709 (3)	0.22705 (16)	0.39932 (7)	0.0467 (4)	
H7	0.7643	0.1920	0.3750	0.056*	
C14	0.7746 (3)	0.30247 (15)	0.64273 (7)	0.0426 (4)	
C20	−0.0950 (3)	−0.00424 (15)	0.61356 (7)	0.0482 (4)	
H20	−0.1941	−0.0273	0.5842	0.058*	
C13	0.9558 (3)	0.36222 (18)	0.67486 (8)	0.0549 (5)	
H13	0.9679	0.3520	0.7120	0.066*	
C23'	0.3812 (3)	0.35939 (16)	0.80082 (7)	0.0490 (4)	
H23'	0.2976	0.3228	0.7704	0.059*	
C3'	0.0937 (3)	0.17794 (17)	1.10136 (7)	0.0475 (4)	
H3'	0.1941	0.1386	1.1199	0.057*	
C6	0.6984 (3)	0.21946 (16)	0.45486 (7)	0.0459 (4)	
H6	0.8114	0.1798	0.4678	0.055*	
C2'	−0.0545 (3)	0.24060 (15)	1.13040 (6)	0.0418 (4)	
C7'	−0.2004 (3)	0.30002 (17)	1.10305 (7)	0.0508 (4)	
H7'	−0.2990	0.3433	1.1225	0.061*	
C6'	−0.1999 (3)	0.29510 (17)	1.04629 (7)	0.0505 (4)	
H6'	−0.2996	0.3354	1.0280	0.061*	
C3	0.3662 (3)	0.33868 (17)	0.41563 (7)	0.0496 (4)	
H3	0.2545	0.3793	0.4026	0.060*	
C23	0.2027 (3)	0.06416 (16)	0.70203 (7)	0.0496 (4)	
H23	0.3013	0.0861	0.7316	0.059*	
C10	0.9248 (3)	0.39496 (16)	0.56390 (8)	0.0479 (4)	
H10	0.9171	0.4069	0.5269	0.057*	
C22	0.0102 (3)	−0.00373 (17)	0.70930 (8)	0.0565 (5)	
H22	−0.0203	−0.0280	0.7437	0.068*	
C13'	−0.3877 (3)	0.07889 (16)	0.83011 (7)	0.0481 (4)	
H13'	−0.3815	0.0722	0.7920	0.058*	
C11'	−0.5702 (3)	0.03041 (17)	0.91091 (8)	0.0504 (4)	
H11'	−0.6884	−0.0084	0.9267	0.060*	
C12'	−0.5596 (3)	0.02260 (17)	0.85331 (7)	0.0520 (4)	
H12'	−0.6705	−0.0212	0.8312	0.062*	
C10'	−0.4088 (3)	0.09435 (15)	0.94365 (7)	0.0433 (4)	
H10'	−0.4180	0.0977	0.9816	0.052*	
C11	1.0943 (3)	0.45122 (17)	0.59649 (9)	0.0580 (5)	
H11	1.1999	0.5017	0.5816	0.070*	
C21	−0.1387 (3)	−0.03607 (17)	0.66532 (8)	0.0543 (5)	
H21	−0.2699	−0.0799	0.6710	0.065*	
C20'	0.6349 (3)	0.47103 (17)	0.89157 (8)	0.0534 (4)	
H20'	0.7200	0.5079	0.9217	0.064*	
C21'	0.6986 (3)	0.48325 (18)	0.83907 (8)	0.0613 (5)	
H21'	0.8278	0.5292	0.8337	0.074*	
C12	1.1107 (3)	0.43343 (19)	0.65267 (9)	0.0609 (5)	
H12	1.2284	0.4708	0.6745	0.073*	
C1	0.3082 (4)	0.3402 (2)	0.30222 (8)	0.0734 (6)	
H1A	0.3146	0.3370	0.2630	0.110*	
H1B	0.3109	0.4226	0.3176	0.110*	
H1C	0.1767	0.2957	0.3116	0.110*	
C22'	0.5729 (3)	0.42791 (17)	0.79378 (8)	0.0575 (5)	
H22'	0.6188	0.4373	0.7585	0.069*	
C1'	−0.2057 (4)	0.2910 (2)	1.21743 (8)	0.0775 (7)	
H1D	−0.1800	0.2827	1.2557	0.116*	
H1E	−0.3458	0.2513	1.2054	0.116*	
H1F	−0.2008	0.3749	1.2121	0.116*	

### Atomic displacement parameters (Å^2^)

**Table:**

	*U*^11^	*U*^22^	*U*^33^	*U*^12^	*U*^13^	*U*^23^
S1	0.0432 (2)	0.0465 (3)	0.0395 (2)	−0.00126 (18)	−0.00526 (17)	0.00235 (18)
S1'	0.0431 (2)	0.0492 (3)	0.0362 (2)	−0.00239 (18)	−0.00513 (16)	−0.00047 (17)
O1	0.0586 (8)	0.0671 (9)	0.0408 (7)	0.0062 (6)	0.0047 (5)	0.0143 (6)
O1'	0.0621 (8)	0.0789 (9)	0.0316 (6)	0.0090 (7)	0.0024 (5)	0.0037 (6)
C17	0.0372 (8)	0.0355 (8)	0.0382 (8)	0.0045 (6)	−0.0021 (6)	−0.0011 (6)
C8'	0.0397 (8)	0.0376 (8)	0.0327 (8)	0.0067 (6)	−0.0027 (6)	0.0041 (6)
C18	0.0427 (8)	0.0362 (8)	0.0401 (9)	0.0034 (7)	0.0015 (7)	−0.0002 (7)
C16'	0.0409 (8)	0.0374 (8)	0.0352 (8)	0.0036 (7)	−0.0021 (6)	0.0048 (6)
C9	0.0374 (8)	0.0370 (8)	0.0456 (9)	0.0069 (7)	0.0031 (7)	−0.0035 (7)
C5'	0.0387 (8)	0.0381 (8)	0.0322 (7)	0.0020 (7)	−0.0019 (6)	0.0034 (6)
C17'	0.0386 (8)	0.0354 (8)	0.0343 (8)	0.0037 (6)	−0.0055 (6)	0.0025 (6)
C8	0.0390 (8)	0.0360 (8)	0.0399 (8)	0.0070 (7)	0.0018 (6)	−0.0015 (6)
C18'	0.0424 (8)	0.0377 (8)	0.0383 (8)	0.0026 (7)	−0.0006 (6)	0.0043 (7)
C16	0.0405 (8)	0.0380 (9)	0.0380 (8)	0.0048 (7)	−0.0007 (6)	−0.0001 (6)
C19	0.0445 (9)	0.0352 (8)	0.0411 (9)	0.0059 (7)	0.0004 (7)	−0.0013 (7)
C14'	0.0418 (8)	0.0401 (9)	0.0362 (8)	0.0027 (7)	−0.0050 (6)	0.0038 (7)
C2	0.0430 (9)	0.0386 (9)	0.0406 (9)	−0.0018 (7)	0.0042 (7)	0.0064 (7)
C15'	0.0463 (9)	0.0460 (9)	0.0314 (8)	0.0005 (7)	−0.0043 (6)	0.0038 (7)
C15	0.0469 (9)	0.0473 (10)	0.0373 (8)	0.0038 (7)	−0.0034 (7)	0.0002 (7)
C4'	0.0432 (9)	0.0561 (10)	0.0381 (9)	0.0164 (8)	0.0033 (7)	0.0070 (7)
C5	0.0390 (8)	0.0347 (8)	0.0386 (8)	0.0033 (6)	0.0036 (6)	0.0015 (6)
C19'	0.0444 (9)	0.0380 (9)	0.0420 (9)	0.0037 (7)	0.0008 (7)	0.0017 (7)
C4	0.0477 (10)	0.0571 (11)	0.0468 (10)	0.0209 (8)	0.0104 (7)	0.0035 (8)
C9'	0.0391 (8)	0.0374 (8)	0.0365 (8)	0.0046 (7)	−0.0023 (6)	0.0045 (6)
C7	0.0491 (10)	0.0481 (10)	0.0445 (9)	0.0137 (8)	0.0104 (7)	0.0016 (7)
C14	0.0372 (8)	0.0418 (9)	0.0470 (9)	0.0044 (7)	−0.0011 (7)	−0.0044 (7)
C20	0.0467 (9)	0.0414 (9)	0.0533 (10)	−0.0001 (7)	−0.0016 (8)	−0.0039 (8)
C13	0.0462 (10)	0.0618 (12)	0.0528 (11)	0.0027 (9)	−0.0070 (8)	−0.0079 (9)
C23'	0.0589 (11)	0.0468 (10)	0.0402 (9)	−0.0012 (8)	0.0021 (8)	0.0055 (7)
C3'	0.0473 (9)	0.0592 (11)	0.0393 (9)	0.0156 (8)	−0.0016 (7)	0.0116 (8)
C6	0.0454 (9)	0.0477 (10)	0.0464 (10)	0.0150 (8)	0.0044 (7)	0.0036 (7)
C2'	0.0444 (9)	0.0470 (10)	0.0323 (8)	−0.0013 (7)	−0.0008 (6)	0.0022 (7)
C7'	0.0535 (10)	0.0595 (11)	0.0403 (9)	0.0213 (9)	0.0029 (7)	−0.0054 (8)
C6'	0.0556 (10)	0.0567 (11)	0.0417 (9)	0.0230 (9)	−0.0063 (8)	0.0023 (8)
C3	0.0461 (9)	0.0562 (11)	0.0504 (10)	0.0171 (8)	0.0031 (8)	0.0123 (8)
C23	0.0557 (10)	0.0489 (10)	0.0424 (9)	−0.0005 (8)	−0.0003 (8)	0.0028 (8)
C10	0.0443 (9)	0.0425 (10)	0.0548 (10)	0.0016 (7)	0.0081 (8)	−0.0041 (8)
C22	0.0700 (13)	0.0509 (11)	0.0473 (10)	−0.0030 (9)	0.0104 (9)	0.0056 (8)
C13'	0.0527 (10)	0.0498 (10)	0.0394 (9)	0.0000 (8)	−0.0094 (7)	0.0017 (7)
C11'	0.0430 (9)	0.0490 (10)	0.0577 (11)	−0.0039 (8)	0.0012 (8)	0.0069 (8)
C12'	0.0479 (10)	0.0497 (10)	0.0546 (11)	−0.0049 (8)	−0.0120 (8)	0.0007 (8)
C10'	0.0456 (9)	0.0434 (9)	0.0405 (9)	0.0020 (7)	0.0022 (7)	0.0047 (7)
C11	0.0439 (10)	0.0475 (11)	0.0772 (14)	−0.0067 (8)	0.0115 (9)	−0.0115 (9)
C21	0.0525 (10)	0.0458 (10)	0.0617 (12)	−0.0067 (8)	0.0103 (9)	0.0003 (9)
C20'	0.0503 (10)	0.0515 (11)	0.0536 (11)	−0.0071 (8)	0.0015 (8)	−0.0051 (8)
C21'	0.0619 (12)	0.0534 (12)	0.0642 (13)	−0.0136 (9)	0.0148 (10)	−0.0004 (9)
C12	0.0405 (10)	0.0631 (13)	0.0722 (14)	−0.0043 (9)	−0.0046 (9)	−0.0160 (10)
C1	0.0792 (15)	0.0909 (17)	0.0565 (12)	0.0178 (13)	−0.0037 (11)	0.0322 (11)
C22'	0.0682 (13)	0.0526 (11)	0.0505 (11)	−0.0058 (9)	0.0151 (9)	0.0076 (9)
C1'	0.0793 (15)	0.112 (2)	0.0405 (11)	0.0155 (14)	0.0159 (10)	−0.0067 (11)

### Geometric parameters (Å, º)

**Table:**

S1—C19	1.7494 (17)	C7—C6	1.377 (2)
S1—C17	1.7540 (15)	C7—H7	0.9300
S1'—C19'	1.7488 (17)	C14—C13	1.422 (2)
S1'—C17'	1.7504 (15)	C20—C21	1.378 (3)
O1—C2	1.3729 (19)	C20—H20	0.9300
O1—C1	1.420 (2)	C13—C12	1.351 (3)
O1'—C2'	1.3727 (18)	C13—H13	0.9300
O1'—C1'	1.421 (2)	C23'—C22'	1.377 (3)
C17—C8	1.379 (2)	C23'—H23'	0.9300
C17—C16	1.422 (2)	C3'—C2'	1.382 (2)
C8'—C17'	1.378 (2)	C3'—H3'	0.9300
C8'—C9'	1.425 (2)	C6—H6	0.9300
C8'—C5'	1.493 (2)	C2'—C7'	1.371 (2)
C18—C23	1.390 (2)	C7'—C6'	1.385 (2)
C18—C19	1.407 (2)	C7'—H7'	0.9300
C18—C16	1.454 (2)	C6'—H6'	0.9300
C16'—C15'	1.371 (2)	C3—H3	0.9300
C16'—C17'	1.421 (2)	C23—C22	1.378 (3)
C16'—C18'	1.451 (2)	C23—H23	0.9300
C9—C8	1.423 (2)	C10—C11	1.363 (2)
C9—C10	1.426 (2)	C10—H10	0.9300
C9—C14	1.428 (2)	C22—C21	1.392 (3)
C5'—C6'	1.385 (2)	C22—H22	0.9300
C5'—C4'	1.389 (2)	C13'—C12'	1.354 (3)
C8—C5	1.492 (2)	C13'—H13'	0.9300
C18'—C23'	1.391 (2)	C11'—C10'	1.361 (2)
C18'—C19'	1.405 (2)	C11'—C12'	1.408 (2)
C16—C15	1.370 (2)	C11'—H11'	0.9300
C19—C20	1.388 (2)	C12'—H12'	0.9300
C14'—C15'	1.402 (2)	C10'—H10'	0.9300
C14'—C13'	1.421 (2)	C11—C12	1.411 (3)
C14'—C9'	1.434 (2)	C11—H11	0.9300
C2—C3	1.374 (2)	C21—H21	0.9300
C2—C7	1.384 (2)	C20'—C21'	1.372 (3)
C15'—H15'	0.9300	C20'—H20'	0.9300
C15—C14	1.407 (2)	C21'—C22'	1.391 (3)
C15—H15	0.9300	C21'—H21'	0.9300
C4'—C3'	1.380 (2)	C12—H12	0.9300
C4'—H4'	0.9300	C1—H1A	0.9600
C5—C4	1.381 (2)	C1—H1B	0.9600
C5—C6	1.396 (2)	C1—H1C	0.9600
C19'—C20'	1.390 (2)	C22'—H22'	0.9300
C4—C3	1.381 (2)	C1'—H1D	0.9600
C4—H4	0.9300	C1'—H1E	0.9600
C9'—C10'	1.418 (2)	C1'—H1F	0.9600
			
C19—S1—C17	91.55 (7)	C12—C13—H13	119.2
C19'—S1'—C17'	91.64 (7)	C14—C13—H13	119.2
C2—O1—C1	116.84 (14)	C22'—C23'—C18'	119.79 (17)
C2'—O1'—C1'	117.75 (15)	C22'—C23'—H23'	120.1
C8—C17—C16	123.04 (14)	C18'—C23'—H23'	120.1
C8—C17—S1	125.08 (12)	C4'—C3'—C2'	119.88 (14)
C16—C17—S1	111.88 (12)	C4'—C3'—H3'	120.1
C17'—C8'—C9'	117.64 (13)	C2'—C3'—H3'	120.1
C17'—C8'—C5'	120.48 (13)	C7—C6—C5	120.82 (15)
C9'—C8'—C5'	121.87 (14)	C7—C6—H6	119.6
C23—C18—C19	119.04 (16)	C5—C6—H6	119.6
C23—C18—C16	129.04 (15)	C7'—C2'—O1'	124.30 (15)
C19—C18—C16	111.91 (14)	C7'—C2'—C3'	119.98 (15)
C15'—C16'—C17'	118.90 (15)	O1'—C2'—C3'	115.73 (14)
C15'—C16'—C18'	129.10 (14)	C2'—C7'—C6'	119.51 (16)
C17'—C16'—C18'	112.00 (13)	C2'—C7'—H7'	120.2
C8—C9—C10	121.87 (16)	C6'—C7'—H7'	120.2
C8—C9—C14	120.09 (15)	C7'—C6'—C5'	121.90 (15)
C10—C9—C14	118.04 (15)	C7'—C6'—H6'	119.0
C6'—C5'—C4'	117.34 (14)	C5'—C6'—H6'	119.0
C6'—C5'—C8'	121.91 (13)	C2—C3—C4	119.38 (15)
C4'—C5'—C8'	120.75 (14)	C2—C3—H3	120.3
C8'—C17'—C16'	123.19 (14)	C4—C3—H3	120.3
C8'—C17'—S1'	125.00 (11)	C22—C23—C18	119.95 (16)
C16'—C17'—S1'	111.81 (12)	C22—C23—H23	120.0
C17—C8—C9	117.52 (14)	C18—C23—H23	120.0
C17—C8—C5	120.25 (14)	C11—C10—C9	121.17 (18)
C9—C8—C5	122.22 (14)	C11—C10—H10	119.4
C23'—C18'—C19'	119.03 (16)	C9—C10—H10	119.4
C23'—C18'—C16'	128.98 (15)	C23—C22—C21	120.23 (17)
C19'—C18'—C16'	112.00 (14)	C23—C22—H22	119.9
C15—C16—C17	118.98 (15)	C21—C22—H22	119.9
C15—C16—C18	129.06 (15)	C12'—C13'—C14'	121.58 (16)
C17—C16—C18	111.94 (13)	C12'—C13'—H13'	119.2
C20—C19—C18	121.03 (16)	C14'—C13'—H13'	119.2
C20—C19—S1	126.27 (13)	C10'—C11'—C12'	120.47 (17)
C18—C19—S1	112.68 (13)	C10'—C11'—H11'	119.8
C15'—C14'—C13'	121.48 (14)	C12'—C11'—H11'	119.8
C15'—C14'—C9'	119.94 (14)	C13'—C12'—C11'	119.97 (16)
C13'—C14'—C9'	118.58 (15)	C13'—C12'—H12'	120.0
O1—C2—C3	124.11 (15)	C11'—C12'—H12'	120.0
O1—C2—C7	115.89 (15)	C11'—C10'—C9'	121.62 (16)
C3—C2—C7	120.00 (15)	C11'—C10'—H10'	119.2
C16'—C15'—C14'	120.69 (14)	C9'—C10'—H10'	119.2
C16'—C15'—H15'	119.7	C10—C11—C12	120.43 (18)
C14'—C15'—H15'	119.7	C10—C11—H11	119.8
C16—C15—C14	120.67 (15)	C12—C11—H11	119.8
C16—C15—H15	119.7	C20—C21—C22	121.10 (17)
C14—C15—H15	119.7	C20—C21—H21	119.5
C3'—C4'—C5'	121.38 (15)	C22—C21—H21	119.5
C3'—C4'—H4'	119.3	C21'—C20'—C19'	118.71 (17)
C5'—C4'—H4'	119.3	C21'—C20'—H20'	120.6
C4—C5—C6	117.68 (15)	C19'—C20'—H20'	120.6
C4—C5—C8	120.80 (14)	C20'—C21'—C22'	121.02 (18)
C6—C5—C8	121.50 (14)	C20'—C21'—H21'	119.5
C20'—C19'—C18'	121.00 (16)	C22'—C21'—H21'	119.5
C20'—C19'—S1'	126.44 (13)	C13—C12—C11	120.16 (17)
C18'—C19'—S1'	112.55 (13)	C13—C12—H12	119.9
C3—C4—C5	122.00 (15)	C11—C12—H12	119.9
C3—C4—H4	119.0	O1—C1—H1A	109.5
C5—C4—H4	119.0	O1—C1—H1B	109.5
C10'—C9'—C8'	122.62 (14)	H1A—C1—H1B	109.5
C10'—C9'—C14'	117.76 (14)	O1—C1—H1C	109.5
C8'—C9'—C14'	119.62 (14)	H1A—C1—H1C	109.5
C6—C7—C2	120.11 (15)	H1B—C1—H1C	109.5
C6—C7—H7	119.9	C23'—C22'—C21'	120.46 (18)
C2—C7—H7	119.9	C23'—C22'—H22'	119.8
C15—C14—C13	121.72 (16)	C21'—C22'—H22'	119.8
C15—C14—C9	119.66 (14)	O1'—C1'—H1D	109.5
C13—C14—C9	118.61 (16)	O1'—C1'—H1E	109.5
C21—C20—C19	118.60 (16)	H1D—C1'—H1E	109.5
C21—C20—H20	120.7	O1'—C1'—H1F	109.5
C19—C20—H20	120.7	H1D—C1'—H1F	109.5
C12—C13—C14	121.57 (18)	H1E—C1'—H1F	109.5
			
C19—S1—C17—C8	−178.68 (14)	C6—C5—C4—C3	0.5 (3)
C19—S1—C17—C16	1.36 (12)	C8—C5—C4—C3	−177.81 (16)
C17'—C8'—C5'—C6'	110.27 (19)	C17'—C8'—C9'—C10'	−178.49 (14)
C9'—C8'—C5'—C6'	−70.9 (2)	C5'—C8'—C9'—C10'	2.7 (2)
C17'—C8'—C5'—C4'	−70.3 (2)	C17'—C8'—C9'—C14'	1.4 (2)
C9'—C8'—C5'—C4'	108.46 (18)	C5'—C8'—C9'—C14'	−177.45 (13)
C9'—C8'—C17'—C16'	−1.2 (2)	C15'—C14'—C9'—C10'	179.21 (15)
C5'—C8'—C17'—C16'	177.68 (14)	C13'—C14'—C9'—C10'	−0.2 (2)
C9'—C8'—C17'—S1'	178.44 (11)	C15'—C14'—C9'—C8'	−0.7 (2)
C5'—C8'—C17'—S1'	−2.7 (2)	C13'—C14'—C9'—C8'	179.93 (14)
C15'—C16'—C17'—C8'	0.2 (2)	O1—C2—C7—C6	−179.65 (15)
C18'—C16'—C17'—C8'	−179.42 (14)	C3—C2—C7—C6	0.0 (3)
C15'—C16'—C17'—S1'	−179.48 (12)	C16—C15—C14—C13	178.99 (16)
C18'—C16'—C17'—S1'	0.93 (17)	C16—C15—C14—C9	−0.5 (2)
C19'—S1'—C17'—C8'	179.39 (14)	C8—C9—C14—C15	−1.0 (2)
C19'—S1'—C17'—C16'	−0.96 (12)	C10—C9—C14—C15	178.29 (15)
C16—C17—C8—C9	−1.9 (2)	C8—C9—C14—C13	179.57 (15)
S1—C17—C8—C9	178.17 (11)	C10—C9—C14—C13	−1.2 (2)
C16—C17—C8—C5	179.16 (13)	C18—C19—C20—C21	−1.0 (2)
S1—C17—C8—C5	−0.8 (2)	S1—C19—C20—C21	−179.45 (13)
C10—C9—C8—C17	−177.15 (14)	C15—C14—C13—C12	−178.89 (17)
C14—C9—C8—C17	2.1 (2)	C9—C14—C13—C12	0.6 (3)
C10—C9—C8—C5	1.8 (2)	C19'—C18'—C23'—C22'	0.8 (3)
C14—C9—C8—C5	−178.99 (14)	C16'—C18'—C23'—C22'	−178.97 (17)
C15'—C16'—C18'—C23'	−0.2 (3)	C5'—C4'—C3'—C2'	0.1 (3)
C17'—C16'—C18'—C23'	179.39 (16)	C2—C7—C6—C5	0.6 (3)
C15'—C16'—C18'—C19'	−179.90 (16)	C4—C5—C6—C7	−0.8 (3)
C17'—C16'—C18'—C19'	−0.36 (19)	C8—C5—C6—C7	177.49 (16)
C8—C17—C16—C15	0.5 (2)	C1'—O1'—C2'—C7'	5.7 (3)
S1—C17—C16—C15	−179.53 (12)	C1'—O1'—C2'—C3'	−174.10 (18)
C8—C17—C16—C18	179.50 (14)	C4'—C3'—C2'—C7'	−0.9 (3)
S1—C17—C16—C18	−0.55 (16)	C4'—C3'—C2'—O1'	178.84 (16)
C23—C18—C16—C15	−1.0 (3)	O1'—C2'—C7'—C6'	−178.77 (17)
C19—C18—C16—C15	178.00 (16)	C3'—C2'—C7'—C6'	1.0 (3)
C23—C18—C16—C17	−179.85 (16)	C2'—C7'—C6'—C5'	−0.2 (3)
C19—C18—C16—C17	−0.85 (19)	C4'—C5'—C6'—C7'	−0.6 (3)
C23—C18—C19—C20	2.4 (2)	C8'—C5'—C6'—C7'	178.79 (17)
C16—C18—C19—C20	−176.73 (14)	O1—C2—C3—C4	179.32 (16)
C23—C18—C19—S1	−179.00 (13)	C7—C2—C3—C4	−0.3 (3)
C16—C18—C19—S1	1.89 (17)	C5—C4—C3—C2	0.1 (3)
C17—S1—C19—C20	176.66 (15)	C19—C18—C23—C22	−1.6 (3)
C17—S1—C19—C18	−1.87 (12)	C16—C18—C23—C22	177.35 (17)
C1—O1—C2—C3	−5.2 (3)	C8—C9—C10—C11	179.81 (15)
C1—O1—C2—C7	174.46 (17)	C14—C9—C10—C11	0.6 (2)
C17'—C16'—C15'—C14'	0.6 (2)	C18—C23—C22—C21	−0.5 (3)
C18'—C16'—C15'—C14'	−179.88 (15)	C15'—C14'—C13'—C12'	−178.44 (16)
C13'—C14'—C15'—C16'	179.04 (15)	C9'—C14'—C13'—C12'	1.0 (3)
C9'—C14'—C15'—C16'	−0.4 (2)	C14'—C13'—C12'—C11'	−0.9 (3)
C17—C16—C15—C14	0.7 (2)	C10'—C11'—C12'—C13'	0.1 (3)
C18—C16—C15—C14	−178.09 (15)	C12'—C11'—C10'—C9'	0.7 (3)
C6'—C5'—C4'—C3'	0.7 (3)	C8'—C9'—C10'—C11'	179.25 (15)
C8'—C5'—C4'—C3'	−178.74 (16)	C14'—C9'—C10'—C11'	−0.6 (2)
C17—C8—C5—C4	66.3 (2)	C9—C10—C11—C12	0.7 (3)
C9—C8—C5—C4	−112.60 (19)	C19—C20—C21—C22	−1.1 (3)
C17—C8—C5—C6	−111.91 (18)	C23—C22—C21—C20	1.9 (3)
C9—C8—C5—C6	69.2 (2)	C18'—C19'—C20'—C21'	1.0 (3)
C23'—C18'—C19'—C20'	−1.2 (2)	S1'—C19'—C20'—C21'	179.79 (15)
C16'—C18'—C19'—C20'	178.60 (15)	C19'—C20'—C21'—C22'	−0.4 (3)
C23'—C18'—C19'—S1'	179.85 (13)	C14—C13—C12—C11	0.7 (3)
C16'—C18'—C19'—S1'	−0.37 (18)	C10—C11—C12—C13	−1.3 (3)
C17'—S1'—C19'—C20'	−178.14 (16)	C18'—C23'—C22'—C21'	−0.2 (3)
C17'—S1'—C19'—C18'	0.76 (13)	C20'—C21'—C22'—C23'	0.0 (3)

### Hydrogen-bond geometry (Å, º)

Cg2, Cg5 and Cg15 are the centroids of the C2–C7, C18–C23 and C9'–C14' rings, respectively.

**Table:**

*D*—H···*A*	*D*—H	H···*A*	*D*···*A*	*D*—H···*A*
C6′—H6′···S1′^i^	0.93	2.87	3.7591 (17)	160
C3′—H3′···*Cg*15^ii^	0.93	2.93	3.704 (2)	141
C7—H7···*Cg*5^iii^	0.93	2.89	3.672 (2)	143
C11—H11···*Cg*2^iv^	0.93	2.91	3.789 (2)	159

Symmetry codes: (i) *x*−1, *y*, *z*; (ii) −*x*, −*y*, −*z*+2; (iii) −*x*+1, −*y*, −*z*+1; (iv) −*x*+2, −*y*+1, −*z*+1.
